# Identification of ciliated sensory neuron-expressed genes in *Caenorhabditis elegans *using targeted pull-down of poly(A) tails

**DOI:** 10.1186/gb-2005-6-2-r17

**Published:** 2005-01-31

**Authors:** Hirofumi Kunitomo, Hiroko Uesugi, Yuji Kohara, Yuichi Iino

**Affiliations:** 1 Molecular Genetics Research Laboratory, The University of Tokyo, 7-3-1 Hongo, Bunkyo-ku, Tokyo 113-0033, Japan; 2 Genome Biology Laboratory, National Institute of Genetics, Mishima 411-8540, Japan

## Abstract

It is not always easy to apply microarray technology to small numbers of cells because of the difficulty in selectively isolating mRNA from such cells. We report here the preparation of mRNA from ciliated sensory neurons of *Caenorhabditis elegans *using the mRNA-tagging method, in which poly(A) RNA was co-immunoprecipitated with an epitope-tagged poly(A)-binding protein specifically expressed in sensory neurons. Subsequent cDNA microarray analyses led to the identification of a panel of sensory neuron-expressed genes.

## Abstract 2

An mRNA-tagging method was used to selectively isolate mRNA from a small number of cells for subsequent cDNA microarray analysis. The approach was used to identify genes specifically expressed in ciliated sensory neurons of *Caenorhabditis elegans*.

## Background

Recent advances in technologies for analyzing whole-genome gene-expression patterns have provided a wealth of information on the complex transcriptional regulatory networks and changes in gene-expression patterns that are related to phenotypic changes caused by environmental stimuli or genetic alterations. Changes in gene expression are also fundamental during development and cellular differentiation, and differences in gene expression lead to different cell fates and eventually determine the structural and functional characteristics of each cell type. Comparative analyses of gene-expression patterns in various cell types will therefore provide a framework for understanding the molecular architecture of these cells as cellular systems.

*Caenorhabditis elegans *is an ideal model organism for investigating development and differentiation at high resolution, because adult hermaphrodites only have 959 somatic nuclei, whose cell lineages are all known. About 19,000 genes were identified by determination of the *C. elegans *genome sequence [1]. Functional genomic approaches, including systematic inhibition of gene functions by RNA interference [2-5], large-scale identification of interacting proteins [6], systematic generation of deletion mutants [7-9], and determination of the time and place of transcription [10-12], are currently in progress to accumulate information on all genes in the genome.

Genome-wide gene-expression profiling using DNA or oligonucleotide microarray technology has also been applied to this organism. Microarrays containing more than 90% of *C. elegans *genes have been constructed and used in global gene-expression analyses under a wide variety of developmental, environmental and genetic conditions [13-15]. Genome-wide gene expression analyses of the germline have also been carried out [16,17]. Mutants lacking functional gonads and those with masculinized or feminized gonads were used in these studies to identify germline-expressed genes and genes correlated with the germline sexes.

To analyze gene-expression patterns in various cells, particularly those forming small tissues, selective isolation of mRNA from these cells is necessary. As an example of this approach, mRNA was prepared from mechanosensory neurons after cell culture of their embryonic precursors followed by selection of the cells by flow cytometry [18]. Although embryonic cell cultures allow the collection of cells at early stages of development, methods for the separation, culture and collection of fully developed tissues have not been established and might be technically difficult.

*C. elegans *modifies its behavior by sensing environmental cues such as food, chemicals, temperature or pheromones. These cues are recognized by approximately 50 sensory neurons positioned in the head and tail. Although the overall functions of the chemosensory or thermosensory neurons have been examined by laser-killing experiments, the molecular mechanisms that underlie the functions of each sensory neuron have not yet been fully explored. Profiling of genes that are expressed in sensory neurons might therefore provide insights into the genes required for the specific functions of neurons.

To identify sensory neuron-expressed genes, we adopted the mRNA-tagging method [19]. In this method, poly(A)-binding protein (PABP), which binds the poly(A) tails of mRNA, is utilized to specifically pull-down poly(A) RNA from the target tissues. By employing this method, we successfully identified novel genes that are expressed in the ciliated sensory neurons of *C. elegans*.

## Results

### Preparation of mRNA from particular types of neurons using mRNA tagging

**Figure 1:**
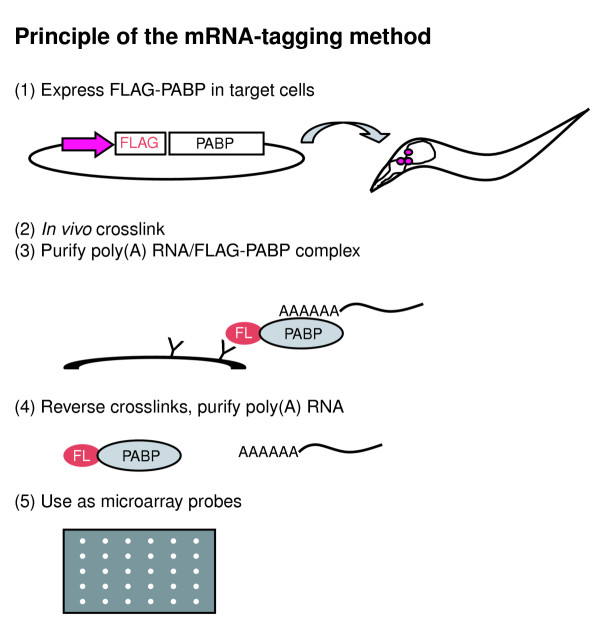
Principle of the mRNA-tagging method. Step 1, FLAG-tagged poly(A)-binding protein (PABP) is expressed from a transgene using a cell-specific promoter. Step 2, PABP and poly(A)^+ ^RNA are crosslinked *in situ *by formaldehyde. Step 3, poly(A)-RNA/FLAG-PABP complexes are purified by anti-FLAG affinity purification. Step 4, RNA-PABP crosslinks are reversed and RNA is isolated. Step 5, purified RNA is used for microarray analysis.

To isolate sensory neuron-expressed transcripts, we devised a method that utilizes PABP. This approach involves the generation of transgenic animals that express an epitope-tagged PABP using cell-specific promoters. Since PABP binds the poly(A) tails of mRNA [20], *in situ *crosslinking of RNA and proteins, followed by affinity purification of the tagged PABP from lysates of these animals, is expected to co-precipitate all the poly(A)^+ ^RNA from cells expressing the tagged PABP (Figure 1). This method was independently devised by Roy *et al*. and used to identify muscle-expressed genes [19], but whether the procedure was applicable to smaller tissues, such as neurons, was unknown. We applied this technology, mRNA tagging [19], to the ciliated sensory neurons of *C. elegans*; these comprise approximately 50 cells whose cell bodies are typically 2 μm in diameter compared to the approximate animal body length of 1 mm.

PABP is encoded by the *pab-1 *gene in *C. elegans*. Nematode strains expressing FLAG-tagged PAB-1 from transgenes were generated using tissue-specific promoters. To prepare mRNA from sensory neurons, we generated the JN501 strain (hereafter called *che-2*::PABP) in which the transgene was expressed in most of the ciliated sensory neurons using a *che-2 *gene promoter [21]. A second strain, JN502 (*acr-5*::PABP), was generated to prepare mRNA from another subset of neurons using an *acr-5 *promoter, which is active in B-type motor neurons, as well as unidentified head and tail neurons [22]. A third strain, JN503 (*myo-3*::PABP), which expressed the transgene in non-pharyngeal muscles using the *myo-3 *promoter [23], was generated to serve as a non-neuronal control. Expression of FLAG-PAB-1 was confirmed by western blotting analyses, and immunohistochemistry using an anti-FLAG antibody (data not shown). Expression patterns were essentially the same as those reported for the promoters used, but we note that expression of FLAG-PAB-1 in ventral cord motor neurons was weak in the *acr-5*::PABP strain compared to that in sensory neurons in the *che-2*::PABP strain. As a measure of the functional integrity of FLAG-PAB-1-expressing cells, responses of the *che-2*::PABP strain to the volatile repellent 1-octanol, which is sensed by ASH amphid sensory neurons was tested. The sensitivity of the *che-2*::PABP animals was indistinguishable from the wild type (data not shown). The ability of the exposed sensory neurons to absorb the lipophilic dye diQ was also tested. Amphid sensory neurons in the head stained normally, whereas phasmid neurons, PHA and PHB, in the tail showed weak defects in dye-filling (90% staining of PHA and 91% staining of PHB, compared to 100% in wild type for both neurons). The *acr-5*::PABP and *myo-3*::PABP strains appeared to move normally, suggesting overall functional integrity of motor neurons and body-wall muscles, respectively.

**Figure 2:**
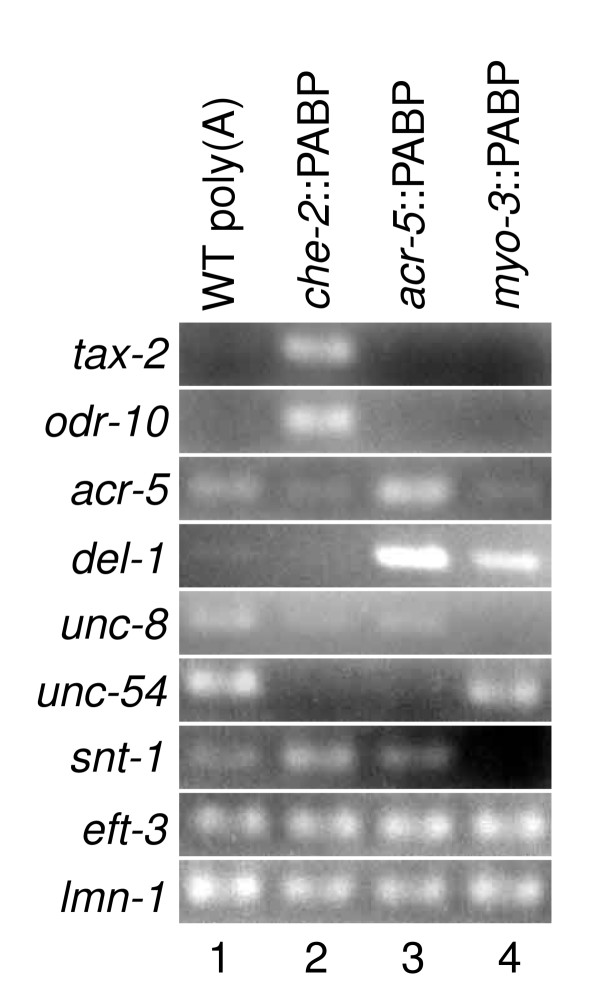
Quantification of tissue-specific transcripts in RNA prepared by mRNA tagging. The transcript indicated on the left of each row was amplified by RT-PCR using gene-specific primers. Poly(A)^+^RNA from wild-type (WT) animals was used as a template in lane 1. RNA prepared by mRNA tagging from *che-2*::PABP (JN501), *acr-5*::PABP (JN502) and *myo-3*::PABP (JN503) was used in lanes 2, 3 and 4, respectively.

Poly(A) RNA/FLAG-PAB-1 complexes were pulled-down from whole lysates of these transgenic worms using anti-FLAG monoclonal antibodies. Poly(A) RNA was then extracted and concentrated. The amounts of known tissue-specific transcripts were examined by reverse transcription PCR (RT-PCR) (Figure 2). The mRNA for *tax-2*, which is expressed in a subset of sensory neurons [24], was enriched in RNA from *che-2*::PABP. The mRNA for *odr-10*, which is expressed in only one pair of sensory neurons [25], was also highly enriched in *che-2*::PABP. On the other hand, mRNA for *acr-5 *and *del-1*, both of which are expressed in B-type motor neurons [22], was enriched in RNA from *acr-5*::PABP. The mRNA for *unc-8*, which is expressed in motor neurons and ASH and FLP sensory neurons in the head [26], was contained in RNA from both *che-*2::PABP and *acr-5*::PABP. The mRNA for *unc-54*, which is expressed in muscles [23], was enriched in RNA from *myo-3*::PABP. Representatives of housekeeping genes, *eft-3 *[27] and *lmn-1 *[28], were detected in RNA from all transgenic strains. Quantitative RT-PCR was performed to estimate the relative amounts of neuron type-specific transcripts. The amount of the *odr-10 *transcript in RNA from *che-2*::PABP was 39-fold higher than that from *acr-5*::PABP, and mRNA for *gcy-6*, which is expressed in only a single sensory neuron [29], was enriched 10-fold. On the other hand, the mRNA for *acr-5 *was enriched eightfold in RNA from *acr-5*::PABP compared with that from *che-2*::PABP. mRNA for the pan-neuronally expressed gene *snt-1 *[30] was equally represented in RNA from both *acr-5*::PABP and *che-2*::PABP. Therefore, selective enrichment of sensory neuron-, motor neuron- and muscle-expressed genes in RNA from *che-*2::PABP, *acr-5*::PABP and *myo-3*::PABP strains, respectively, have been achieved as intended. Of these, the enrichment of motor neuron-expressed genes appeared less efficient, because weak bands were sometimes seen for these genes in RT-PCR from *che-*2::PABP or *myo-3*::PABP RNA.

### cDNA microarray experiments

We used a cDNA microarray to compare the properties of mRNA prepared from *che-*2-expressing ciliated sensory neurons with that from *acr-5*-expressing cells. RNA purified from *che-2*::PABP was labeled with Cy5 and that from *acr-5*::PABP was labeled with Cy3. The two types of labeled RNA were mixed and hybridized to the cDNA microarray and the *che-2*::PABP/*acr-5*::PABP (Cy5/Cy3) ratio was calculated for each cDNA spot. The cDNA microarray contained 8,348 cDNA spots corresponding to 7,088 *C. elegans *genes. Two sets of independently prepared RNA samples were hybridized to two separate arrays. The logarithm of the hybridization intensity ratio for each spot, log_2_(*che-2*::PABP/*acr-5*::PABP), was calculated and values from the two experiments were averaged. This calculation allowed us to order the genes represented on the microarrays according to the log_2_(*che-2*::PABP/*acr-5*::PABP) value (see Additional data file 1). Genes specifically expressed in *che-2*-expressing cells should have higher rank orders in this list, whereas those expressed in *acr-5*-expressing cells should have lower rank orders.

**Figure 3:**
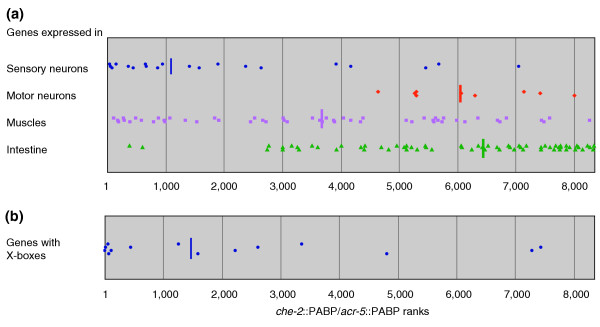
Rank orders of *che-2*::PABP/*acr-5*::PABP values for specific genes in the microarray analyses. **(a) **Distribution of genes with known expression patterns. Genes known to be specifically expressed in sensory neurons, motor neurons, muscles or the intestine, respectively, were collected from WormBase (see Materials and methods) and the rank orders of their *che-2*::PABP/*acr-5*::PABP signal ratios were plotted. Vertical bars indicate the medians. Genes expressed in sensory neurons are specifically enriched in the *che-2*::PABP RNA preparations, while motor neuron- and intestine-expressed genes are enriched in the *acr-5*::PABP RNA preparations. Note that although only five genes were found as motor neuron-expressed genes, nine data points were plotted in (a), because multiple cDNA clones were present on the microarray for three of the genes (see Additional data file 2). **(b) **Distribution of genes with X-boxes in their promoter regions. Genes that carry one or more X-boxes in their promoter regions were collected from the genome database (see Materials and methods) and their rank orders of *che-2*::PABP/*acr-5*::PABP signal ratios were plotted. These genes, which are expected to be expressed in ciliated sensory neurons under the control of the DAF-19 transcription factor, are also enriched in the *che-2*::PABP RNA preparations.

To evaluate the results of the microarray experiments, we searched for genes that are known to be expressed in amphid sensory neurons, but not in ventral cord motor neurons, or vice versa, using the WormBase database (WS94). Of these, 20 sensory neuron-specific genes and five motor neuron-specific genes were present on the arrays (see Additional data files 1 and 2). These genes showed a highly uneven distribution, with sensory neuron-specific genes concentrated in the highest rank orders and motor neuron-specific genes distributed in lower rank orders (Figure 3a). Muscle-expressed genes (also found using WormBase) were almost evenly distributed. However, intestine-expressed genes were concentrated in the lower rank orders. These results demonstrate that our mRNA isolation procedure specifically enriched ciliated sensory neuron- and motor neuron-expressed genes as intended. The unexpected distribution of the intestine-expressed genes will be discussed later.

*daf-19 *encodes a transcription factor similar to mammalian RFX2. Several genes expressed in ciliated sensory neurons and essential for ciliary morphogenesis, such as *che-2 *and *osm-6*, are under the control of *daf-19 *and have one or more copies of the *cis*-regulatory element X-box in their promoter regions [31]. We therefore examined the distribution of genes that harbor X-boxes in their promoter regions. Again, the distribution of X-box-containing genes was highly uneven (Figure 3b, see also Additional data files 1 and 2), further demonstrating the successful enrichment of ciliated neuron-expressed genes.

### Expression analysis of candidate sensory neuron-expressed genes by reporter fusions

The above analyses showed that sensory neuron-expressed genes were enriched in the mRNA population purified from *che-2*::PABP. However, only a few genes were previously known to be expressed in these tissues. In fact, the expression patterns for most top-ranked genes in our list were not known. To determine which of these genes were actually expressed in sensory neurons, we examined the expression patterns of 17 genes with the highest rank orders using translational green fluorescent protein (GFP) fusions. The expression patterns for these genes had not been reported previously.

**Figure 4:**
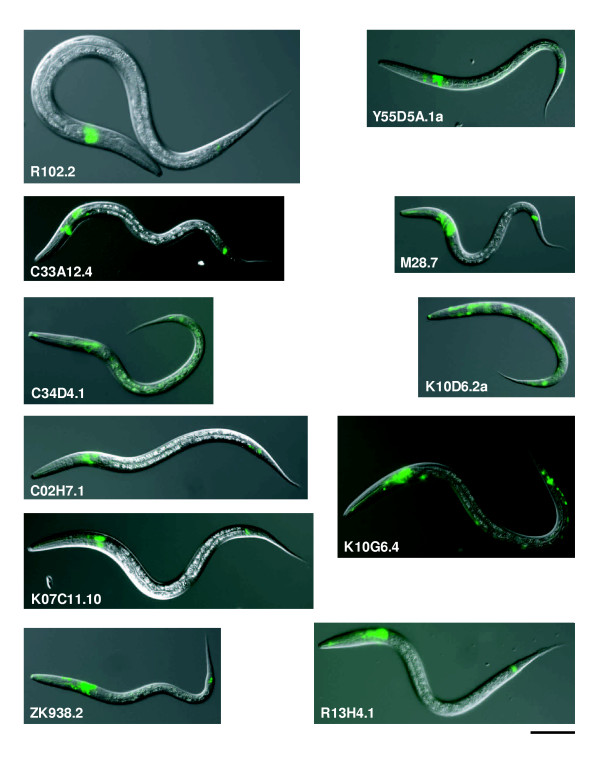
Expression patterns of newly identified sensory neuron-expressed genes. The genes indicated were each fused to GFP in-frame, and the reporters introduced into wild-type animals. Overlaid images of the Nomarski and GFP fluorescence images of transgenic worms between larval stages 1 and 3 are shown. Gene expression is indicated by the green fluorescence. Scale bar, 50 μm. See Table 1 for the identity of the expressing cells.

We did not observe any GFP fluorescence for two clones, K07B1.8 and C13B9.1, probably because the promoter region we selected did not contain all the functional units or expression was below the level of detection. GFP-expressing cells were identified for all the remaining 15 genes (Figure 4, Table 1). For 13 of these GFP fusions, expression was observed in ciliated sensory neurons, namely amphid, labial and/or phasmid sensory neurons. Of these, expression in the intestine, in addition to the sensory neurons, was observed for Y55D5A.1a and T07C5.1c, whereas expression of K10D6.2a was also observed in seam cells and the main body hypodermis (hyp7). Expression of K10G6.4 was observed in many other neurons in addition to sensory neurons. Expression in the intestine and coelomocytes, but not in sensory neurons, was observed for two other clones, C35E7.11 and F10G2.1, respectively. In summary, of the 15 genes whose expression patterns could be determined, 13 (87%) were expressed in sensory neurons. These results showed that most of the genes with the highest rank orders were expressed in ciliated sensory neurons.

We also examined the expression patterns of two genes with the lowest rank orders (Y44A6D.2 and T08A9.9/*spp-5*). Expression in the ventral nerve cord was observed for Y44A6D.2, while only weak expression in the intestine was observed for T08A9.9 (data not shown). These results also suggested that our procedure was somewhat less effective in enriching motor neuron-expressed genes than sensory neuron-expressed genes (Figure 3a).

### Categorization of *che-2*::PABP-enriched genes reveals specific features

**Figure 5:**
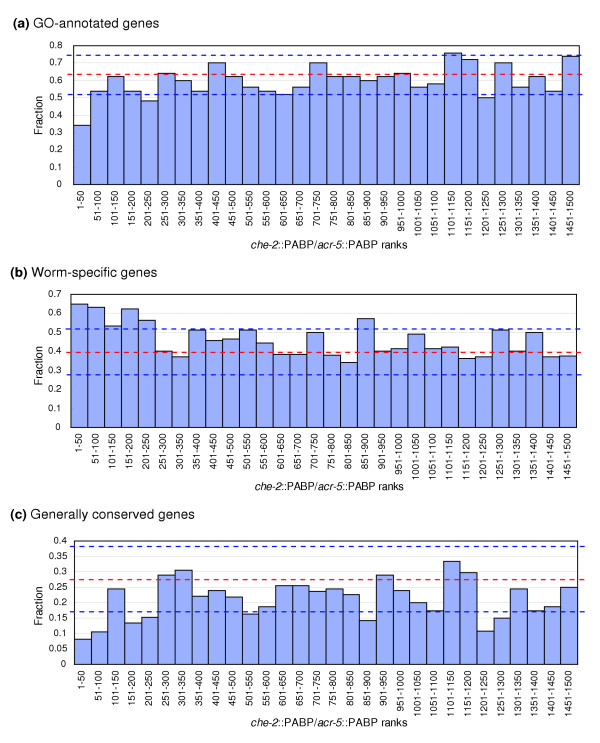
Sensory neuron-specific genes are less likely to be classified into Gene Ontology categories and more likely to be worm-specific. **(a) **All genes on the microarray were ordered by descending *che-2*::PABP/*acr-5*::PABP value and the fraction of GO-annotated genes in each bin is indicated for a bin width of 50 rank orders. Only the top 1,500 genes are shown in (a)-(c). **(b) **The fraction of genes with homologs in *C. briggsae*, and not in humans, mice, flies, fission yeast or budding yeast (cutoff BLASTP score E = 1 × 10^-20^) in each bin is indicated as in (a). **(c) **The fraction of genes with homologs in both animals and yeasts, namely in humans, mice or flies and in fission yeast or budding yeast (cutoff BLASTP score E = 1 × 10^-20^) in each bin is indicated as in (a). In all panels, the red dotted line indicates the average of all the genes, and the blue dotted lines indicate the 95% confidence limits assuming a random binominal distribution.

In an attempt to characterize ciliated sensory neuron-expressed genes as a set, we first referred to functional annotations of each gene generated by the WormBase. It was noted that the fraction of genes with functional annotations was smaller for the highest ranked genes (Figure 5a). BLASTP searches of the nonredundant (nr) protein sequence database and proteome datasets for several representative animal and yeast species showed that nematode-specific genes were enriched, while those with homologs in yeast and other animals tended to be under-represented in the top-ranked genes (Figure 5b,c).

**Figure 6:**
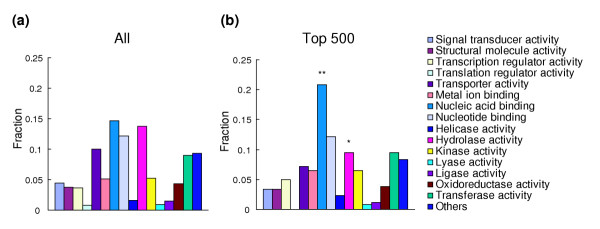
Categories of genes enriched in the sensory neuron fraction. Genes were categorized according to the GO molecular function categories. **(a) **Categorization of all the genes on the microarray; **(b) **categorization of genes within the top 500 *che-2*::PABP/*acr-5*::PABP ranks. In both panels, the fraction of genes in each category in respect of all annotated genes is shown.*P < 0.05; **P < 0.01 (binominal distribution).

Among the genes with Gene Ontology (GO) annotations, top-ranked genes showed a significantly larger fraction with a 'nucleic acid binding' functional capacity (*P *= 0.004, Figure 6). Protein motifs found to be enriched among the *che-2*::PABP-enriched genes included 'cuticle collagen', 'chromo domain', 'linker histone' and 'laminin G domain'.

Another prominent characteristic of the *che-2*::PABP-derived mRNA fraction was enrichment of genes homologous to nephrocystins. Nephrocystins are responsible for a hereditary cystic kidney disease, nephronophthisis, and to date, nephrocystin 1 (*NPHP1*) through nephrocystin 4 (*NPHP4*) have been identified [32-35]. *C. elegans *homologs of *NPHP1 *and *NPHP4 *were ranked at positions 15 and 25 in our list, suggesting a link between these disease genes and the functions of worm sensory neurons.

## Discussion

### Preparation of mRNA from a subset of neurons in *C. elegans*

We prepared poly(A) RNA from a subset of neurons using the mRNA-tagging technique. The genome-wide identification of muscle-expressed genes demonstrated that mRNA tagging is a powerful technique for collecting tissue-specific transcripts in *C. elegans *[19]. The method is especially useful in this organism because dissection and separation of the tissues are difficult because of the worm's small size and the presence of cuticles. However, it was not known whether this method was applicable to smaller tissues, such as subsets of neurons. In this study, we attempted to isolate mRNA from ciliated sensory neurons using mRNA tagging. Although the volume of target neurons was much smaller than that of muscles, transcripts of various sensory neuron-expressed genes, ranging from those expressed in many sensory neurons to those expressed in only one or two sensory neurons, were successfully enriched.

The procedure of mRNA tagging is based on immunoprecipitation of poly(A)-RNA/FLAG-PAB-1 complexes. A potential problem with this technique is that once the cells are broken, poly(A) RNA released from non-target cells might bind unoccupied FLAG-PAB-1. To reduce this possibility, we adopted stringent washing conditions in addition to *in situ *formaldehyde crosslinking. Although this procedure reduced the recovery of immunocomplexes, it ensured minimal contamination by mRNA from non-target cells. As there are many characterized promoters that can deliver FLAG-PAB-1 to small numbers of neurons in *C. elegans*, profiling of the gene-expression pattern of each type of neuron should be possible with this technique.

Another potential problem with this method is that PABP might have different binding affinities for different transcript species, rendering some tissue-specific transcripts difficult to recover. Although PABP binds tightly to the poly(A) tails of most mRNA [36], RNA species co-immunoprecipitated with PABP from cultured cells do not represent the total RNA of the cells [37]. This might also cause another problem in that transcripts with strong PABP affinity might be undesirably enriched in the precipitates and cause unexpected biases.

### Analysis of purified mRNA using a cDNA microarray

Preparation and characterization of EST clones led to the identification of more than 10,000 cDNA groups corresponding to different genes of *C. elegans *([12,38] and Y.K., unpublished results). We used a cDNA microarray on which such cDNA clones were spotted to identify the genes expressed in ciliated sensory neurons. Using a cDNA microarray rather than a genome DNA microarray has the advantage that genes on the array have guaranteed expression, and hybridization to the corresponding mRNA species is efficient. The microarray we used contained 7,088 genes of *C. elegans*, representing 40% of the predicted genes on the genome [1]. On the other hand, there are also genes that were not represented in our cDNA collection, including characterized sensory neuron-specific genes such as *osm-6 *[39] and most seven-transmembrane receptor genes including *odr-10*; this might be a disadvantage of using a cDNA microarray. Acquisition of cDNA clones for rare mRNA species and use of whole-genome microarrays are complementary approaches for improving the applicability of the method described here.

### Evaluation of microarray experiments

Previously known sensory neuron- and motor neuron-expressed genes were used to evaluate the results of our microarray analyses. Most genes were enriched in our *che-2*::PABP-derived mRNA preparations or in the *acr-5*::PABP-derived mRNA preparations depending on their expression patterns. However, several genes were not enriched as expected. Furthermore, enrichment of motor neuron-expressed genes in the *acr-5*::PABP-derived mRNA preparations appeared less efficient. The reasons for these occurrences are unknown, but the expression of FLAG-PAB-1 in motor neurons were low in the *acr-5*::PABP strain, which could account for the low efficiency of enrichment for this tissue. Another potential problem is that the expression pattern of the *acr-5 *promoter has not been fully characterized [22], and both the *che-2 *and *acr-5 *promoters are active in labial neurons, where expression of the *acr-5 *promoter was relatively strong compared to motor neurons (data not shown). Genes expressed in the intestine were enriched in the *acr-5*::PABP-derived mRNA preparations. FLAG-PAB-1 was weakly expressed in both the *che-2*::PABP and *acr-5*::PABP strains in intestine, with the latter showing higher level of expression (data not shown). Low-level expression of artificially manufactured genes in the intestine seems to be quite common, either due to readthrough transcription from the vector or the 3' regulatory sequences. Our results may suggest that in future applications one must be very careful about this type of low-level expression of FLAG-PAB-1.

We determined the expression patterns of genes highly enriched in the *che-2*::PABP-derived mRNA preparations. Thirteen of 15 genes that showed clear expression patterns of GFP reporters were expressed in multiple sensory neurons. None of these genes has previously been characterized. In addition, quantitative PCR analysis shows that genes expressed in only one or two neurons, *gcy-6 *and *odr-10*, respectively, can be enriched. Therefore, our procedure is effective for identifying genes that are preferentially expressed in a particular subset of cells. On the other hand, the presence of small fractions of genes that are predominantly expressed in tissues other than sensory neurons was also evident. Therefore, mRNA-tagging technology should be regarded as enrichment of candidate cell-specific genes and the real expression pattern of each gene should be verified independently.

### Characterization of the sensory neuron-expressed gene set

Since most of the genes enriched in the *che-2*::PABP-derived mRNA preparations proved to be sensory neuron-expressed, characterization of the enriched genes as a set should lead to molecular characterization of the sensory neurons of *C. elegans*. A prominent feature of the genes enriched in the *che-2*::PABP-derived mRNA preparations is that they include nematode-specific genes more often than the rest of the genes, as judged from inter-species BLASTP comparisons, suggesting that many of the genes identified have functions unique to nematode sensory neurons. The existence of many nematode-specific gene families has previously been noted, and was proposed to be related to the nematode-specific body plan [40]. Since we were obviously counter-selecting for ubiquitously expressed genes that serve common cellular functions, a lower representation of highly conserved genes is expected. In addition, these observations indicate that our approach is effective for identifying hitherto uncharacterized genes that might be important for specific functions of differentiated cell types.

Identification of panels of genes expressed in particular cells will also be useful for understanding the regulatory network of gene expression. In this context, it is of interest to examine whether we can identify *cis*-acting elements commonly found in the promoter regions of the sensory neuron-expressed genes. The enrichment of X-boxes in the *che-2*::PABP fraction suggests that this might be plausible. In addition, other reports have identified *cis*-acting elements in genes expressed during particular developmental stages or in particular neurons (see, for example [41,42]). However, searches for common sequences using the MEME program did not reveal any motifs that were enriched in the *che-2*::PABP fraction. This is likely to be due to the heterogeneity of our sensory neuron-expressed gene collection (see the expression patterns in Table 1). Further refinement of our gene sets by expression analysis of each gene will be required to identify *cis*-acting elements that regulate cell-specific gene expression.

By surveying GO annotations and protein motifs, genes whose predicted functions are related to nucleic acids and/or chromatin were found to be enriched in the *che-2*::PABP gene set. This might indicate that *C. elegans *sensory neurons have specialized regulatory mechanisms for gene expression, although it remains to be seen which of these 'chromatin' genes are actually expressed in a sensory neuron-specific manner. It was also apparent from visual inspection or computer searches that two homologs of nephrocystins are included in the highest rank orders. It has recently been shown that nephrocystin 1 and nephrocystin 4 interact with each other and are both components of cilia. These studies have led to the hypothesis that the kidney disease nephronophthisis is caused by malfunctions of cilia on the tubular epithelium [33-35,43]. *C. elegans *ciliated sensory neurons also have prominent ciliary structures [44], but none of the other cell types in this organism has any cilia. It has also been found that all *C. elegans *homologs (*bbs-1, 2, 7 *and *8*) of the human genes responsible for Bardet-Biedl syndrome, which is also thought to be a ciliary disease, are specifically expressed in ciliated sensory neurons [45]. It is therefore likely that the gene set revealed by our analysis includes *C. elegans *homologs of as yet unidentified ciliary disease genes.

## Conclusions

The present study demonstrates that a combination of mRNA tagging and microarray analysis is an effective strategy for identifying genes expressed in subsets of neurons. Systematic reporter expression analyses following this approach will facilitate the accumulation of information regarding gene expression patterns. In particular, profiling of the gene expression patterns of subsets of neurons, in combination with analyses of neural functions, might provide insights into understanding the distinct roles of cells within the neural network.

## Materials and methods

### Generation of strains expressing FLAG-PAB-1 in a tissue-specific manner

The initiation codon of a cDNA for *pab-1*, yk28d10, was replaced with a linker composed of two complementary oligonucleotides, 5'-AATTGCTAGCATGGATTACAAGGATGATGACGATAAGT-3' and 5'-CTAGACTTATCGTCATCATCCTTGTAATCCATGCTAGC-3', in which the underlined sequence encodes an initiation codon followed by a FLAG peptide. The resulting epitope-tagged gene was cloned into the pPD49.26 vector (donated by Andy Fire, Stanford University). The promoter of *che-2 *[21], *acr-5 *[22] or *myo-3 *[23] was inserted 5' upstream to the fusion gene to generate the FLAG-PAB-1 expression plasmids pche2-FLAG-PABP(FL), pacr5-FLAG-PABP(FL) and pmyo3-FLAB-PABP(FL), respectively. Wild-type animals were transformed with each expression construct, along with the pRF4 plasmid, which carries a dominant *rol-6 *allele, as a marker [46]. Stable integrated transgenic strains were generated from unstable transgenic lines as described [47]. Each integrated strain was outcrossed twice with wild-type N2. The genotypes of these strains were: JN501: *Is *[*che-2*p::*flag*-*pab-1 *pRF4]; JN502: *Is *[*acr-5*p::*flag*-*pab-1 *pRF4]; and JN503: *Is *[*myo-3*p::*flag*-*pab-1 *pRF4].

### mRNA tagging

To purify poly(A)-RNA/FLAG-PAB-1 complexes from subsets of neurons, we modified a protocol for chromosome immunoprecipitation [48]. Transgenic animals were grown in liquid as described previously [49]. The worms were then harvested and washed twice with M9 [50]. To crosslink poly(A) RNA with FLAG-PAB-1 *in vivo*, worms were treated with 1% formaldehyde in M9 for 15 min at 20°C with gentle agitation. The formaldehyde was then inactivated by 125 mM glycine for 5 min at 20°C and washed out by replacing the buffer with four changes of TBS (20 mM Tris-HCl pH 7.5, 150 mM NaCl). At this point, worms were dispensed into 0.4 g aliquots, placed in 2-ml microtubes and stored frozen until lysate preparation.

Worms were resuspended in 0.45 ml lysis buffer (50 mM HEPES-KOH pH 7.3, 1 mM EDTA, 140 mM KCl, 10% glycerol, 0.5% Igepal CA-630 (Sigma), 1 mM DTT, 0.2 mM PMSF, protease inhibitor cocktail (Complete-EDTA, Roche) at the recommended concentration) supplemented with 20 mM ribonucleoside vanadyl complexes (RVC, Sigma) and 1000 U/ml of human placental ribonuclease inhibitor (Takara). Animals were disrupted by vigorous shaking with 2 g acid-washed glass beads (Sigma), and worm debris was removed by centrifugation at 18,000 *g *for 20 min. Five hundred microliters of supernatant, with the protein concentration roughly adjusted to 20 mg/ml, was incubated with 50 μl of anti-FLAG M2 affinity gel beads (Sigma) for 2 h. The affinity beads were sequentially washed three times with lysis buffer supplemented with PMSF, twice with wash buffer (50 mM HEPES-KOH pH 7.3, 1 mM EDTA, 1 M KCl, 10% glycerol, 0.5% Igepal CA-630, 1 mM DTT) and once with TE (10 mM Tris-HCl pH 7.5, 0.5 mM EDTA). Lysate preparation and purification of RNA-protein complexes were performed at 4°C. Precipitated materials were eluted with 100 μl elution buffer (50 mM Tris-HCl pH 7.5, 10 mM EDTA, 1% SDS, 20 mM RVC) by incubation for 5 min at 65°C. Elution was repeated and the two supernatant fractions were combined. The eluted RNA/FLAG-PAB-1 complexes were incubated for 6 h at 65°C to reverse the formaldehyde crosslinks. Proteins were digested with proteinase K and removed by phenol-chloroform extraction. Nucleic acid was recovered by ethanol precipitation. Typically, 100 ng nucleic acid was obtained from 0.5 ml cleared lysate of *myo-3*::PABP. Under the above washing conditions, binding of free poly(A) RNA to PABP was severely impaired (data not shown).

### Examination of the functional integrity of FLAG-PAB-1-expressing cells in the *che-2*::PABP strain

For staining of living animals with lipophilic dye, we followed the procedure described before [51] except that diQ (Molecular Probes) was used instead of FITC. Forty-six wild type and 56 *che-2*::PABP worms at L4 to young adult were observed. Cells were identified by their positions and the percentage of stained cells was scored. Responses of the *che-2*::PABP strain to 1-octanol was assessed as described [52] except that Eppendorf Microloader (Eppendorf) was used to deliver 1-octanol to animals' noses.

### RT-PCR

Fifty nanograms of RNA was converted to cDNA using an RNA PCR Kit (AMV) Ver. 2.1 (Takara) according to the manufacturer's protocol. One-tenth of the cDNA from each sample was subjected to a gene-specific PCR reaction in a total volume of 20 μl. Quantification of the PCR products was performed using a FastStart DNA Master SYBR Green I Kit (Roche) with the Light Cycler system (Roche). Serial dilutions of cDNA prepared from poly(A) RNA of wild-type worms were used to generate a standard curve. The ratio of expression levels for each gene was calculated using the amount of *eft-3 *as a reference, and the results of three independent experiments were averaged. The primers used for the amplification of each gene were: lmn1-52: 5'-CGTTCACCACCCACCAGAA-3' and lmn1-32: 5'-CAAGACGAGCTGATGGGTTATCT-3' for *lmn-1*; eft3-52: 5'-ATTGCCACACCGCTCACA-3' and eft3-32: 5'-CCGGTACGACGGTCAACCT-3' for *eft-3*; tax2-54: 5'-GATTAATCCAAGACAAGTTCCTAAATTGAT-3' and tax2-34: 5'-TTCAATTCTTGAACTCCTTTGTTTTC-3' for *tax-2*; unc8-52: 5'-TCTCAGATTTTGGAGGTAATATTGGA-3', and unc8-32: 5'-GATCTCGCAGAAAAGTTCTGCAA-3' for *unc-8*; unc54-52: 5'-AACAGAAGTTGAAGACCCAGAAGAA-3', and unc54-32: 5'-TGGTGGGTGAGTTGCTTGTACT-3' for *unc-54*; snt1-51: 5'-GAGCTGAGGCATTGGATGGA-3' and snt1-31: 5'-CCAAGTGTATGCCATTGAGCAA-3' for *snt-1*; acr5-52: 5'-AATCGATTTATGGACAGAATTTGGA-3' and acr5-32: 5'-ATGTTGCAAAAGAAGTGGGTCTAGA-3' for *acr-5*; odr10-51: 5'-TCATTGTGTTTTGCTCATTTCTGTAC-3' and odr10-31: 5'-ATATTGTTCTTCGGAAATCACGAAT-3' for *odr-10*; del1-51: 5'-TAAACTGCCTCACGACAGAAG-3' and del1-31: 5'-GCCATCAAGTTGAACCAAGAAT-3' for *del-1*. All primers were designed to include one intron in the PCR product amplified from the genomic DNA for each gene, such that the length and melting point were different from the product amplified from the cDNA. In Figure 2, *eft-3 *was amplified for 25 cycles, *lmn-1*, *snt-1 *and *unc-54 *for 30 cycles and *tax-2*, *unc-8*, *odr-10, del-1 *and *acr-5 *for 35 cycles. Amplified DNA was visualized by electrophoresis followed by staining with ethidium bromide.

### cDNA microarray analysis

Microarrays were prepared using a 16-pin arrayer constructed according to the format of Patrick Brown (Stanford University [53]) on CMT-GAPS-coated glass slides. Two micrograms of RNA prepared from JN501 was reverse-transcribed using oligo(dT) primers and SuperScript II reverse transcriptase (Lifetech) with the addition of Cy5-dCTP to generate Cy5-labeled probes. RNA prepared from JN502 was similarly used for the generation of Cy3-labeled probes. Equal amounts of the two probes were mixed and hybridized to a single array overnight at 42°C in Gene TAC Hyb Buffer (Genomic Solutions). Each array was then washed in 1× SSC/0.03% SDS at 42°C, followed by successive washes in 0.2× SSC and 0.05× SSC at room temperature. The fluorescence intensity of each spot was scanned using a ScanArray Lite (Perkin Elmer) and analyzed by QuantArray (GSI Lumonics).

### Reporter constructs for determination of expression patterns

A genomic DNA fragment for each gene was amplified by PCR such that it contained an upstream promoter region followed by a partial or full-length predicted coding region. The 3' PCR primers were designed to introduce a restriction site in-frame with GFP in the vectors. The 5' PCR primers were designed to anneal to a sequence 0.6-5.0 kilobases (kb) upstream of the predicted coding region of each gene. The upstream-predicted gene was essentially not included in the promoter fragment. The amplified PCR fragments were cloned into pPD95.70, pPD95.75 (donated by A. Fire) or a Gateway vector (Invitrogen) to create GFP fusions (see Additional data file 3 for details). The resulting reporter plasmid for each gene was introduced into wild-type animals. Transgenic worms at all developmental stages were observed under a differential interference contrast (DIC)-fluorescence microscope. Cells were identified according to their positions [54] by comparing the fluorescence images of GFP and DiQ staining with Nomarski images of the same animal. At least two independent transgenic lines were observed to confirm the expression patterns. Images were obtained as described previously [17].

### Bioinformatics

cDNA clones were mapped to the *C. elegans *genome using BLAT [55] and BLASTN programs, corresponding gene models were identified in the WormBase annotations [56] and protein sequences were obtained from WormPep122. The gene ontology annotation dataset for *C. elegans *was obtained from the Gene Ontology Consortium [57].

For Figure 3, genes known to be expressed in specific tissues were searched for using the expression pattern search interface of WormBase [58] or using the AcePerl AceDB server [59]. The gene set 'Sensory neurons' was defined as genes expressed in all or some ciliated sensory neurons (including amphid neurons), but not in motor neurons or the ventral nerve cord, according to WormBase. The gene set 'Motor neurons' was genes expressed in VB or DB ventral cord motor neurons and no more than one type of ciliated sensory neuron, or those expressed in cholinergic neurons. The gene set 'Muscles' was genes expressed in some muscles, but not in neurons or the intestine, while 'Intestine' was genes expressed in the intestine, but not in neurons or muscles. X-boxes were searched for using MEME and MAST [60] based on the definition matrix deduced from Swoboda *et al*. [31]. Only between 60 and 160 base-pairs (bp) upstream of the initiation codon of each gene were considered, since Swoboda *et al*. observed that X -boxes were present about 100 bp upstream of the initiation codons [31].

The proteome data set for *Caenorhabditis briggsae*, whose draft genome sequence has recently been released, was downloaded from WormBase [61]. Proteome data sets for humans, mice, *Drosophila melanogaster*, *Schizosaccharomyces pombe *and *Saccharomyces cerevisiae *were obtained from NCBI [62], and BLASTP searches were performed using the WormPep protein sequence as a query for each *C. elegans *gene.

Protein motifs that preferentially appeared in genes at the higher rank orders were searched for as follows. For all genes represented in our microarrays, the protein motifs contained in each gene product were obtained from the AcePerl server. For each motif, deviation of the average log_2_(*che-2*::PABP/*acr-5*::PABP) value for all genes that carried the motif was calculated. To avoid artifactual results due to gene families with close sequence similarities (such as major sperm proteins), groups of genes whose mRNA are expected to cross-hybridize were treated as a single imaginary gene with the average log_2_(*che-2*::PABP/*acr-5*::PABP) value.

## Additional data files

The following additional data are available with the online version of this article. Additional data file 1 is a table listing the results of the microarray experiments. Additional data file 2 lists genes expressed in sensory neurons, motor neurons, muscles and the intestine, and those with X-boxes shown in Figure 3. Additional data file 3 lists the primers and vectors used for reporter constructions. Additional data file 4 contains the legends to the above three tables.

## Supplementary Material

Additional data file 1 a table listing the results of the microarray experiments Click here for additional data file

Additional data file 2 Genes expressed in sensory neurons, motor neurons, muscles and the intestine, and those with X-boxes shown in Figure 3 Click here for additional data file

Additional data file 3 The primers and vectors used for reporter constructions Click here for additional data file

Additional data file 4 The legends to the above three tables Click here for additional data file

## Figures and Tables

**Table 1:** Expression patterns of the top-ranked genes

Rank	Clone	Gene	Locus	Expression pattern
1	yk380a6	**R102.2**		**ADF, ADL, ASH, ASI, ASJ, ASK, PHA, PHB**
2	yk305a7	**C33A12.4**		**ADF, ADL, ASE, ASH, ASI, ASJ, ASK, **AVJ**, AWA, AWB, PHA, PHB, labial neurons**
3	yk139b4	**C34D4.1**		**ADL, ASH, ASI, ASJ, ASK, PHA**
4	yk534e12			
5	yk91d12	**C02H7.1**		**ADF, ADL, AFD, ASG, ASH, ASI, ASJ, ASK, AWB, PHA, PHB, URX**
6	yk261h1	Y43F8C.4		
7	yk538c3	**K07C11.10**		**ADF, ADL, ASE, ASG, ASH, ASI, ASJ, ASK, AWA, AWB, AWC, PHA, PHB**
8	yk561g1	F40H3.6		
9	yk267a7	**ZK938.2**		**ADL, ASE, ASG, ASH, ASI, ASJ, ASK, AWB, AWC, PHB, URX**
10	yk509b4	**Y55D5A.1a**		**ADF, ADL, AFD, ASE, ASG, ASH, ASI, ASJ, ASK, AWA, AWB, AWC, BAG, PHA, PHB, URX, **intestine
11	yk609e11	T27E4.3	*hsp-16.48*	
12	yk561g1	F40H3.6		
13	yk341h9	**F53A9.4**		**ADL, ASE, ASH, ASI, ASJ, ASK, AWC, PHA, PHB, labial neurons**
14	yk604g4	**C35E7.11**		Intestine, RMF, RMH
15	yk467b4	**M28.7**		**ADF, ADL, AFD, ASE, ASG, ASH, ASI, ASJ, ASK, AWA, AWB, AWC, PHA, PHB, URX, labial neurons**
16	yk284g4	**K10D6.2a**		**ADL, ASE, ASI, ASJ, ASK, PHB, URX, labial neurons, **seam cells, hypodermis
17	yk610e5	Y9D1A.1		
18	yk295d7	**K07B1.8**		No GFP
19	yk252h2	C29H12.3a	*rgs-3*	
20	yk225f3	C27A7.4	*che-11*	
21	yk488h9	**C13B9.1**		No GFP
22	yk373g4	**T07C5.1c**		**AFD, ASG, **AUA, PVQ, intestine
23	yk305c8	**F10G2.1**		Coelomocytes
24	yk450c2	**K10G6.4**		**ADF, ADL, AFD, ASE, ASG, ASH, ASI, ASJ, ASK, **AVA, AVD, **AWA, AWB, AWC, PHB, **RMD, ventral nerve cord neurons, many other neurons
25	yk76f1	**R13H4.1**		**ADF, ADL, ASE, ASG, ASH, ASI, ASJ, ASK, PHA, PHB, URX, labial neurons**

The expression patterns of the genes indicated in bold were examined. Only the cells and tissues in which GFP expression was consistently observed are listed. It is therefore possible that the genes are weakly expressed in cells or tissues other than those listed here. Cells and cell groups in bold are ciliated sensory neurons.

## References

[B1] The *C. elegans *Sequencing Consortium (1998). Genome sequence of the nematode *C. elegans *: a platform for investigating biology.. Science.

[B2] Fraser AG, Kamath RS, Zipperlen P, Martinez-Campos M, Sohrmann M, Ahringer J (2000). Functional genomic analysis of *C. elegans *chromosome I by systematic RNA interference.. Nature.

[B3] Gonczy P, Echeverri C, Oegema K, Coulson A, Jones SJ, Copley RR, Duperon J, Oegema J, Brehm M, Cassin E (2000). Functional genomic analysis of cell division in *C. elegans *using RNAi of genes on chromosome III.. Nature.

[B4] Kamath RS, Fraser AG, Dong Y, Poulin G, Durbin R, Gotta M, Kanapin A, Le Bot N, Moreno S, Sohrmann M (2003). Systematic functional analysis of the *Caenorhabditis elegans *genome using RNAi.. Nature.

[B5] Maeda I, Kohara Y, Yamamoto M, Sugimoto A (2001). Large-scale analysis of gene function in *Caenorhabditis elegans *by high-throughput RNAi.. Curr Biol.

[B6] Li S, Armstrong CM, Bertin N, Ge H, Milstein S, Boxem M, Vidalain PO, Han JD, Chesneau A, Hao T (2004). A map of the interactome network of the metazoan *C. elegans*.. Science.

[B7] Jansen G, Hazendonk E, Thijssen KL, Plasterk RH (1997). Reverse genetics by chemical mutagenesis in *Caenorhabditis elegans*.. Nat Genet.

[B8] Edgley M, D'Souza A, Moulder G, McKay S, Shen B, Gilchrist E, Moerman D, Barstead R (2002). Improved detection of small deletions in complex pools of DNA.. Nucleic Acids Res.

[B9] Gengyo-Ando K, Mitani S (2000). Characterization of mutations induced by ethyl methanesulfonate, UV, and trimethylpsoralen in the nematode *Caenorhabditis elegans*.. Biochem Biophys Res Commun.

[B10] McKay SJ, Johnsen R, Khattra J, Asano J, Baillie DL, Chan S, Dube N, Fang L, Goszczynski B, Ha E (2003). Gene expression profiling of cells, tissues, and developmental stages of the nematode *C. elegans*.. Cold Spring Harb Symp Quant Biol.

[B11] Dupuy D, Li QR, Deplancke B, Boxem M, Hao T, Lamesch P, Sequerra R, Bosak S, Doucette-Stamm L, Hope IA (2004). A first version of the Caenorhabditis elegans promoterome.. Genome Res.

[B12] NEXTDB (The Nematode Expression Pattern Database). http://nematode.lab.nig.ac.jp.

[B13] Jiang M, Ryu J, Kiraly M, Duke K, Reinke V, Kim SK (2001). Genome-wide analysis of developmental and sex-regulated gene expression profiles in *Caenorhabditis elegans*.. Proc Natl Acad Sci USA.

[B14] Kim SK, Lund J, Kiraly M, Duke K, Jiang M, Stuart JM, Eizinger A, Wylie BN, Davidson GS (2001). A gene expression map for *Caenorhabditis elegans*.. Science.

[B15] Romagnolo B, Jiang M, Kiraly M, Breton C, Begley R, Wang J, Lund J, Kim SK (2002). Downstream targets of *let-60 Ras *in *Caenorhabditis elegans*.. Dev Biol.

[B16] Reinke V, Smith HE, Nance J, Wang J, Van Doren C, Begley R, Jones SJ, Davis EB, Scherer S, Ward S (2000). A global profile of germline gene expression in *C. elegans*.. Mol Cell.

[B17] Hanazawa M, Mochii M, Ueno N, Kohara Y, Iino Y (2001). Use of cDNA subtraction and RNA interference screens in combination reveals genes required for germ-line development in *Caenorhabditis elegans*.. Proc Natl Acad Sci USA.

[B18] Zhang Y, Ma C, Delohery T, Nasipak B, Foat BC, Bounoutas A, Bussemaker HJ, Kim SK, Chalfie M (2002). Identification of genes expressed in *C. elegans *touch receptor neurons.. Nature.

[B19] Roy PJ, Stuart JM, Lund J, Kim SK (2002). Chromosomal clustering of muscle-expressed genes in *Caenorhabditis elegans*.. Nature.

[B20] Gallie DR (1998). A tale of two termini: a functional interaction between the termini of an mRNA is a prerequisite for efficient translation initiation.. Gene.

[B21] Fujiwara M, Ishihara T, Katsura I (1999). A novel WD40 protein, CHE-2, acts cell-autonomously in the formation of *C. elegans *sensory cilia.. Development.

[B22] Winnier AR, Meir JY, Ross JM, Tavernarakis N, Driscoll M, Ishihara T, Katsura I, Miller DM (1999). UNC-4/UNC-37-dependent repression of motor neuron-specific genes controls synaptic choice in *Caenorhabditis elegans*.. Genes Dev.

[B23] Okkema PG, Harrison SW, Plunger V, Aryana A, Fire A (1993). Sequence requirements for myosin gene expression and regulation in *Caenorhabditis elegans*.. Genetics.

[B24] Coburn CM, Bargmann CI (1996). A putative cyclic nucleotide-gated channel is required for sensory development and function in *C. elegans*.. Neuron.

[B25] Sengupta P, Chou JH, Bargmann CI (1996). *odr-10 *encodes a seven transmembrane domain olfactory receptor required for responses to the odorant diacetyl.. Cell.

[B26] Tavernarakis N, Shreffler W, Wang S, Driscoll M (1997). *unc-8*, a DEG/ENaC family member, encodes a subunit of a candidate mechanically gated channel that modulates *C. elegans *locomotion.. Neuron.

[B27] Mitrovich QM, Anderson P (2000). Unproductively spliced ribosomal protein mRNAs are natural targets of mRNA surveillance in *C. elegans*.. Genes Dev.

[B28] Liu J, Ben-Shahar TR, Riemer D, Treinin M, Spann P, Weber K, Fire A, Gruenbaum Y (2000). Essential roles for *Caenorhabditis elegans *lamin gene in nuclear organization, cell cycle progression, and spatial organization of nuclear pore complexes.. Mol Biol Cell.

[B29] Yu S, Avery L, Baude E, Garbers DL (1997). Guanylyl cyclase expression in specific sensory neurons: a new family of chemosensory receptors.. Proc Natl Acad Sci USA.

[B30] Nonet ML, Grundahl K, Meyer BJ, Rand JB (1993). Synaptic function is impaired but not eliminated in *C. elegans *mutants lacking synaptotagmin.. Cell.

[B31] Swoboda P, Adler HT, Thomas JH (2000). The RFX-type transcription factor DAF-19 regulates sensory neuron cilium formation in *C. elegans*.. Mol Cell.

[B32] Hildebrandt F, Otto E, Rensing C, Nothwang HG, Vollmer M, Adolphs J, Hanusch H, Brandis M (1997). A novel gene encoding an SH3 domain protein is mutated in nephronophthisis type 1.. Nat Genet.

[B33] Mollet G, Salomon R, Gribouval O, Silbermann F, Bacq D, Landthaler G, Milford D, Nayir A, Rizzoni G, Antignac C (2002). The gene mutated in juvenile nephronophthisis type 4 encodes a novel protein that interacts with nephrocystin.. Nat Genet.

[B34] Olbrich H, Fliegauf M, Hoefele J, Kispert A, Otto E, Volz A, Wolf MT, Sasmaz G, Trauer U, Reinhardt R (2003). Mutations in a novel gene, NPHP3, cause adolescent nephronophthisis, tapeto-retinal degeneration and hepatic fibrosis.. Nat Genet.

[B35] Otto EA, Schermer B, Obara T, O'Toole JF, Hiller KS, Mueller AM, Ruf RG, Hoefele J, Beekmann F, Landau D (2003). Mutations in INVS encoding inversin cause nephronophthisis type 2, linking renal cystic disease to the function of primary cilia and left-right axis determination.. Nat Genet.

[B36] Gorlach M, Burd CG, Dreyfuss G (1994). The mRNA poly(A)-binding protein: localization, abundance, and RNA-binding specificity.. Exp Cell Res.

[B37] Tenenbaum SA, Carson CC, Lager PJ, Keene JD (2000). Identifying mRNA subsets in messenger ribonucleoprotein complexes by using cDNA arrays.. Proc Natl Acad Sci USA.

[B38] Reboul J, Vaglio P, Tzellas N, Thierry-Mieg N, Moore T, Jackson C, Shin-i T, Kohara Y, Thierry-Mieg D, Thierry-Mieg J (2001). Open-reading-frame sequence tags (OSTs) support the existence of at least 17,300 genes in *C. elegans*.. Nat Genet.

[B39] Collet J, Spike CA, Lundquist EA, Shaw JE, Herman RK (1998). Analysis of *osm-6*, a gene that affects sensory cilium structure and sensory neuron function in *Caenorhabditis elegans*.. Genetics.

[B40] Blaxter M (1998). *Caenorhabditis elegans *is a nematode.. Science.

[B41] Beer MA, Tavazoie S (2004). Predicting gene expression from sequence.. Cell.

[B42] Wenick AS, Hobert O (2004). Genomic cis-regulatory architecture and trans-acting regulators of a single interneuron-specific gene battery in *C. elegans*.. Dev Cell.

[B43] Watnick T, Germino G (2003). From cilia to cyst.. Nat Genet.

[B44] Ward S, Thomson N, White JG, Brenner S (1975). Electron microscopical reconstruction of the anterior sensory anatomy of the nematode *Caenorhabditis elegans*.. J Comp Neurol.

[B45] Ansley SJ, Badano JL, Blacque OE, Hill J, Hoskins BE, Leitch CC, Kim JC, Ross AJ, Eichers ER, Teslovich TM (2003). Basal body dysfunction is a likely cause of pleiotropic Bardet-Biedl syndrome.. Nature.

[B46] Mello CC, Kramer JM, Stinchcomb D, Ambros V (1991). Efficient gene transfer in *C. elegans *: extrachromosomal maintenance and integration of transforming sequences.. EMBO J.

[B47] CPC: *C. elegans *protocols. http://cobweb.dartmouth.edu/~ambros/worms/index.html.

[B48] Chu DS, Dawes HE, Lieb JD, Chan RC, Kuo AF, Meyer BJ (2002). A molecular link between gene-specific and chromosome-wide transcriptional repression.. Genes Dev.

[B49] Lewis JA, Fleming JT (1995). Basic culture methods.. Methods Cell Biol.

[B50] Brenner S (1974). The genetics of *Caenorhabditis elegans*.. Genetics.

[B51] Hedgecock EM, Culotti JG, Thomson JN, Perkins LA (1985). Axonal guidance mutants of Caenorhabditis elegans identified by filling sensory neurons with fluorescein dyes.. Dev Biol.

[B52] Chao MY, Komatsu H, Fukuto HS, Dionne HM, Hart AC (2004). Feeding status and serotonin rapidly and reversibly modulate a *Caenorhabditis elegans *chemosensory circuit.. Proc Natl Acad Sci USA.

[B53] Pat Brown's Lab. http://cmgm.stanford.edu/pbrown.

[B54] White J, Southgate E, Thomson J, Brenner S (1986). The structure of the nervous system of the nematode *Caenorhabditis elegans*.. Phil Trans R Soc Lond Ser B.

[B55] Kent WJ (2002). BLAT - the BLAST-like alignment tool.. Genome Res.

[B56] WormBase - bulk downloads (*C. elegans*). ftp://ftp.wormbase.org/pub/wormbase/elegans.

[B57] Gene Ontology Consortium Current Annotations - WormBase. http://www.geneontology.org/cgi-bin/GO/downloadGOGA.pl/gene_association.wb.gz.

[B58] WormBase: expression pattern search. http://www.wormbase.org/db/searches/expr_search.

[B59] WormBase. http://aceserver.cshl.org.

[B60] MEME -introduction. http://meme.sdsc.edu/meme/website.

[B61] WormBase - bulk downloads (*C. briggsae*). ftp://ftp.wormbase.org/pub/wormbase/briggsae.

[B62] NCBI genome assembly/annotation projects. ftp://ftp.ncbi.nih.gov/genomes.

