# Supplementary material for: Identification of ciliated sensory neuron-expressed genes in Caenorhabditis elegans using targeted pull-down of poly(A) tails
Source: Genome Biol. 2005 Jan 31;6(2):R17. doi: 10.1186/gb-2005-6-2-r17 (PMC551537; doi:10.1186/gb-2005-6-2-r17)
Supplement: Additional data file 4 — The legends to the above three tables [file gb-2005-6-2-r17-s4.doc]

**Legends to Supplementary tables.**

Supplementary Table 1 Results of the microarray experiments. All spots on the microarray were ordered by descending *che-2*::PABP/*acr-5*::PABP value. The columns indicate the following from left to right. "Rank", *che-2*::PABP/*acr-5*::PABP rank order. "Spot number", spot ID number on the microarray. "Exp. 1/2 che-2/acr-5 ratio", *che-2*::PABP/*acr-5*::PABP signal ratios for experiments 1 and 2, respectively. "Exp. 1/2 normalized log2 (che-2/acr-5)", log_2_(*che-2*::PABP/*acr-5*::PABP) values for experiments 1 and 2, respectively, which were normalized so that the averages of all spots were zero. "Average normalized log2 (che-2/acr-5)", average of the normalized log_2_(*che-2*::PABP/*acr-5*::PABP) values for experiments 1 and 2. "cDNA name", name of the cDNA spotted on the microarray. "5'-EST, 3'-EST", 5' EST and 3' EST, respectively, if the sequence information is available. "Location", location of the EST sequences mapped to the genome. The chromosome number is followed by the base numbers in the chromosome coordinates for each exon separated by commas. The ranges for 5' EST and 3' EST are separated by semicolons. If more than two possible locations were found, they are separated by slashes. "Cosmid", cosmid or YAC clone on which the EST sequences were mapped. "Corresponding CDS", coding sequence annotation by WormBase that corresponds to the cDNA. "Locus", genetic name for the gene. "Homology", representative homolog found in BLASTP searches of the nr-aa database. If no homologs outside *C. elegans* were found, the best *C. elegans* match, if any, is shown. "Motif", Interpro motifs found in the gene product. "Gene Ontology category", Gene Ontology annotation for the gene product. "WormBase: sensory/muscle/intestine expression", + shows that the gene is described in WormBase as being expressed in the tissue. "X box", + shows that the gene has one or more X boxes in the promoter region. The same data are also shown in Supplementary Table 2. "This study: sensory expression/other expression", + shows the indicated expression patterns as extracted from Table 1.

Supplementary Table 2 List of genes expressed in sensory neurons, motor neurons, muscles and the intestine, and those with X boxes shown in Figure 3. "Rank", "spot number", "cDNA name", "CDS", "locus" are defined as in Supplementary Table 1. "WormBase ID" indicates the ID number for the description of the expression patterns. "Description" indicates the description of the expression patterns in WormBase. See Materials and Methods for the criteria for selecting the genes in each category.

Supplementary Table 3 Primers and vectors used for reporter constructions. Each column indicates the following. "Promoter length", length of the DNA fragment 5’ upstream of the predicted coding region that was used to construct the reporter for each gene. "aa GFP fused to", number of amino acids from the amino terminus to the GFP fusion point. "5’/3’ restriction site", restriction sites introduced into the primers and used to subclone the amplified genomic DNA fragment into the vector.
